# Jejunojejunal Intussusception Induced by a Gastrointestinal Stromal Tumor

**DOI:** 10.1155/2012/173680

**Published:** 2012-11-19

**Authors:** Ali H. Zakaria, Salam Daradkeh

**Affiliations:** Istishari Hospital, The University of Jordan, P.O. Box 13261, Amman 11942, Jordan

## Abstract

*Background*. Adult intussusception is a rare entity representing less than 1% of all intestinal obstructions. Diagnosis of the condition is difficult requiring a high index of suspicion and the utilization of imaging studies, especially CT scans. Diagnostic laparoscopy and/or exploratory laparotomy can be used as a diagnostic and therapeutic intervention. In over 90% of cases, an underlying lead point is identified. In the patient described here, it was a gastrointestinal stromal tumor (GIST), a relatively rare mesenchymal tumor comprising only 0.2–1.0% of the gastrointestinal tract neoplasms and believed to originate from neoplastic transformation of the interstitial cells of Cajal. GISTs may occur anywhere along the gastrointestinal tract, but most commonly arise in the stomach and small intestine. Literature review revealed only few cases reporting GISTs as a leading point of adult's intussusception. *Case Presentation*. In this report, we are presenting a rare case of jejunojejunal intussusception in a 78-year-old female patient with a GIST located in the terminal jejunum being the leading point, demonstrating the importance of imaging studies, especially CT scan, laparoscopy, and exploratory laparotomy as diagnostic and therapeutic interventions.

## 1. Introduction

Intestinal invagination or intussusception is the leading cause of intestinal obstruction in children, but it is an uncommon process in adults, accounting for only 5% of all intussusceptions and 1% of all intestinal obstruction. Unlike childhood intussusception which is idiopathic in 90% of cases, 70–90% of adult cases have a demonstrable lead point, with a well-definable neoplastic abnormality being the etiology in 65% of cases [1, 2].

Adult intussusception may present with acute, subacute, or chronic nonspecific symptoms. Therefore, the initial diagnosis often is missed or delayed till the patient is in the operating room. There is a surgical consensus that adult intussusception requires surgical resection because the majority of patients have intraluminal lesions. However, there is controversy about the need for reduction of the intussusception and the extent of resection to be performed [2].

## 2. Case Report

A seventy-eight-year-old female patient with a previous medical history of diabetes mellitus, hypertension, ischemic heart disease, and Hypothyroidism presented with a one-week history of abdominal pain and nausea with vomiting following meals. Her symptoms progressively worsened to severe abdominal distention, anorexia and obstipation on the day of admission. She had no fever, chills, bleeding per rectum, or previous abdominal surgeries. Four weeks earlier the patient had similar symptoms. A plain abdominal X-ray and CT scan which were done at that time showed dilated small bowel loop in right lower quadrant without detected lesions or significant wall thickening, and she had been treated conservatively. After resolution of her symptoms—during the previous episode—an upper and lower GI endoscopy were done and revealed moderate erosive esophagitis, Helicobacter pylori positive gastritis and duodenitis, and an essentially normal colonic exam.

**Figure 1:**
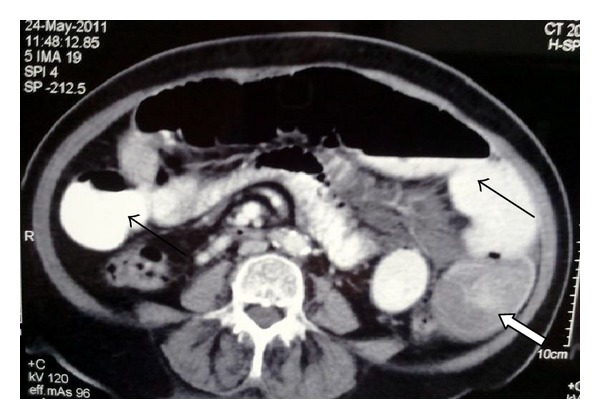
Computed tomography of abdomen showing dilated bowel loops (arrows) and the *target sign *of intussusception (white arrow).

Her vital signs on admission were within normal limits. Examination showed a distended abdomen with hyperactive bowel sounds and generalized tenderness without peritoneal signs. Her laboratory investigations showed anemia with hemoglobin of 7.2 g/dL and white cell count of 9.9 × 10^3^/mm^3^. Abdominal X-ray revealed dilated proximal bowel loops with multiple air fluid levels. Conservative measures with bowel rest, nasogastric intubation, and intravenous fluids failed to control her symptoms. Abdominal and pelvic CT-scan showed markedly dilated fluid filled small bowel loops with one loop showing central hyperdense area, and a thick soft tissue mass around it with multiple small hypodense areas, referred to as the *target sign* [3] (Figure 1).

**Figure 2:**
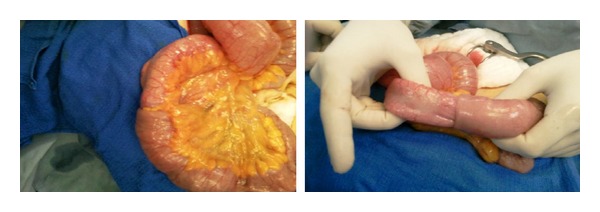
Intraoperative view showing the jejunojejunal intussusception.

**Figure 3:**
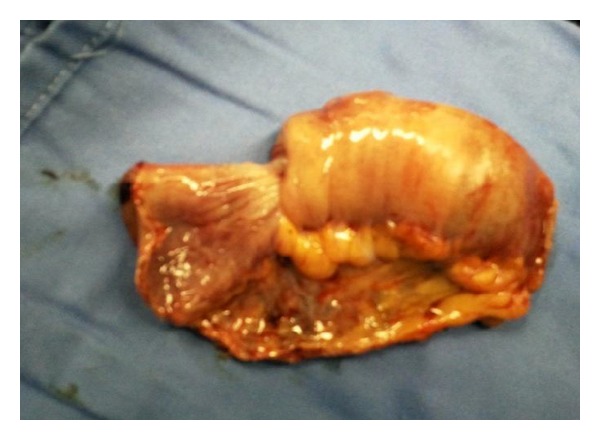
Jejunal tumor (GIST) was the leading point of intussusception.

Diagnostic laparoscopy which revealed a distal jejuno-jejunal intussusception was followed by limited laparotomy and resection then anastomosis was performed (Figure 2). The leading point of the invagination was jejunal tumor (Figure 3). Histopathologic examination of the resected specimen revealed a GIST of intermediate risk, and resection was complete with viable ends. 

The patient had an uneventful postoperative recovery and was discharged in a well condition. She was doing well on subsequent clinic followup.

## 3. Discussion

Adult intussusception is an uncommon clinical entity encountered by surgeons. The exact mechanism is unknown, and it is believed that any lesion in the bowel wall or irritant within the lumen that alters normal peristaltic activity is able to initiate invagination. It is most commonly located at the junctions between freely moving segments and retroperitoneally or adhesionally fixed segments [4].

About 90% of occurrences in adults have a well-defined pathological lead point, which may be a benign—such as benign neoplasms, inflammatory lesions, Meckel's diverticuli, appendix, and adhesions—or malignant lesion. In small intestine, malignant lesions (either primary or metastatic) account for 14–47% of cases, while malignant etiology is more prominent in large bowel representing up to 66% of the cases [2].

Most adult patients with intussusception present with chronic and nonspecific symptoms suggestive of intestinal obstruction. Abdominal pain is the most common symptom followed by nausea, vomiting, and a palpable abdominal mass [1, 2].

Preoperative imaging may help in identifying the causative lesion. Plain abdominal X-rays are typically the first diagnostic tool; with barium studies (showing “stacked coin” or “coiled spring” in upper GI series and “cup-shaped defect” in barium enema), ultrasonography (showing “target and doughnut sign” on transverse view and the “pseudokidney sign” in longitudinal view), and colonoscopy are also useful tools for evaluating intussusception [5–7].

In recent years, with a diagnostic accuracy of 58–100% in recent series, abdominal CT-scan (with the characteristic “*target sign*”) has been reported to be the most useful tool for diagnosis of intestinal intussusception and is regarded superior to the above mentioned studies [3, 8, 9].

Treatment of adult intussusception is always surgical. However, optimal management remains controversial. Most of the debate focuses on the issue of primary resection versus initial reduction followed by a more limited resection [2, 9], keeping in mind that reduction should not be attempted with any degree of suspicion of malignancy, due to possible risks of intraluminal seeding, venous embolization in regions of ulcerated mucosa, and anastomotic complications, which may potentially lead to bowel perforation [10]. Recently, there are several case reports about using laparoscopy as a minimally invasive technique for both diagnosis and treatment of adult intussusceptions.

Mesenchymal tumors constitute only 1% of primary GI cancers with GISTs being the most common. The annual incidence is between 7 and 20 cases per million per year [11]. It occurs predominantly in middle-aged and older individuals and rarely in those under the age of 40. The majority of cases are sporadic; however, several familial cases with heritable mutations in the KIT gene have been identified [12].

GISTs are thought to derive from neoplastic transformation of interstitial cells of Cajal (ICC). They may occur throughout the GI tract from the esophagus to the anus, but most commonly are found in the stomach (40–60%) and jejunum/ileum (25–30%) [12]. Duodenum (5%), colorectum (5–15%), and esophagus (≤1%) are less common sites.

Sometimes GISTs are asymptomatic and are discovered incidentally during an endoscopy or on a CT done for another purpose. More often, they are associated with nonspecific symptoms (i.e., early satiety, bloating) unless complicated with ulceration and overt GI bleeding (40% of the cases) or grow large enough to cause pain, mass or a lead point of intussusception and intestinal obstruction (20% of the cases) [12].

Contrast-enhanced CT is a preferred initial imaging study for screening and staging. However, other procedures such as ultrasound, endoscopy, intestinal capsule, and PET scan may also be used.

Surgical “en bloc” segmental resection with the goal of achieving negative resection margins is the treatment of choice for potentially resectable tumors, while initial therapy with *imatinib* may be preferred if a tumor is borderline resectable, or if resection would necessitate extensive organ disruption.

The prognosis of small intestine GISTs depends upon the adequacy of resection, tumor size, mitotic activity, and location within the small bowel, with small intestine having a worse prognosis than stomach [13–15].

## 4. Conclusion

Intussusception in adults occurs relatively rarely; however, in 90% of cases a specific lead point is identified. The diagnosis may be challenging because of nonspecific symptoms, and sometimes it is impossible to reach the diagnosis of intussusception as a cause of obstruction until laparotomy is done. CT-scan is the most useful imaging modality in diagnosis. An underlying malignant lesion might be the lead point especially in large bowel. Therefore, surgeons should think of intussusception as a cause of intestinal obstruction, and they should be familiar with the various treatment options. The decision whether to undertake resection or reduction followed by resection is case specific, and it should be tailored according to the situation.
